# Supplementary material for: A stress assembly that confers cell viability by preserving ERES components during amino-acid starvation
Source: eLife. 2014 Nov 11;3:e04132. doi: 10.7554/eLife.04132 (PMC4270098; doi:10.7554/eLife.04132)
Supplement: Figure 6—Source data 1. — DOI: http://dx.doi.org/10.7554/eLife.04132.016 [file elife-04132-fig6-data1.pdf]

***Figure 6-figure source data 1*: Low complexity sequences in proteins related to stress granules and P-bodies, and to the secretory pathway.**

A **Stress granules and P-bodies**

Protein	Flybase ID	LCs AA	Total AA	% LC	p-value
Atx2.1	FBgn0041188	489	1084	45.11%	<10-5
Caprin	FBgn0042134	450	961	46.83%	<10-5
FMR1	FBgn0028734	182	729	24.97%	<10-5
Gawki	FBgn0051992	416	1384	30.06%	<10-5
Squid	FBgn0263396	114	344	33.14%	<10-5
TDP43	FBgn0025790	119	531	22.41%	NS
Tral	FBgn0041775	317	657	48.25%	<10-5
Ago	FBgn0041171	221	1326	16.67%	NS
Dcp1	FBgn0034921	17	372	4.57%	<10-5
Rox8	FBgn0005649	141	470	30.00%	NS

B **Secretory pathway**

Protein	Flybase ID	LCs AA	Total AA	% LC	p-value
Sec16	FBgn0052654	580	2021	28.70%	<10-5
Sar1	FBgn0038947	0	193	0.00%	NS
Sec24AB	FBgn0033460	294	1184	24.83%	<10-5
Sec24CD	FBgn0262126	308	1231	25.02%	<10-5
Sec23	FBgn0262125	28	781	3.59%	NS
Sec13	FBgn0024509	49	356	13.76%	NS
Sec31	FBgn0033339	143	1241	11.52%	NS
dGMAP	FBgn0027287	355	1398	25.39%	<10-5
dGRASP	FBgn0036919	101	460	21.96%	<10-5
p125	FBgn0031990	247	2016	12.25%	NS
Garz	FBgn0264560	197	1983	9.93%	NS
Beta COP	FBgn0008635	45	964	4.67%	NS
Alpha COP	FBgn0025725	90	1234	7.29%	NS
Gamma COP	FBgn0028968	15	897	1.67%	NS
Epsilon COP	FBgn0027496	0	306	0.00%	NS
Delta COP	FBgn0028969	88	532	16.54%	<10-2
Erk7	FBgn0052703	106	916	11.57%	NS
KDEL receptor	FBgn0022268	0	212	0.00%	NS
GM130	FBgn0034697	193	795	24.28%	<10-5
Lava lamp	FBgn0029688	595	2779	21.41%	<10-5

NS: non significant. The proteins with an LCS content above 21% are marked in yellow
